# An Ultra-Fast Green UHPLC-MS/MS Method for Assessing the In Vitro Metabolic Stability of Dovitinib: In Silico Study for Absorption, Distribution, Metabolism, Excretion, Metabolic Lability, and DEREK Alerts

**DOI:** 10.3390/medicina60101626

**Published:** 2024-10-04

**Authors:** Mohamed W. Attwa, Ali S. Abdelhameed, Adnan A. Kadi

**Affiliations:** Department of Pharmaceutical Chemistry, College of Pharmacy, King Saud University, P.O. Box 2457, Riyadh 11451, Saudi Arabia; asaber@ksu.edu.sa (A.S.A.); akadi@ksu.edu.sa (A.A.K.)

**Keywords:** Dovitinib, DEREK software, WhichP450 software, pediatric osteosarcoma, ADME profile, UHPLC-MS/MS

## Abstract

*Background and Objectives*: Dovitinib (DVB) is a pan-tyrosine kinase inhibitor (TKI) that can be administered orally. In September 2023, the FDA granted Oncoheroes approval to proceed with an Investigational New Drug (IND) application for dovitinib. This application is intended for the treatment of relapsed or advanced juvenile solid tumors, namely, osteosarcoma. *Materials and Methods*: The target of the present study was to develop a rapid, green, accurate, and sensitive UHPLC-MS/MS method for measuring DVB levels in human liver microsomes (HLMs). The validations of the HLMs were performed via the established UHPLC-MS/MS approach, as stated in the US FDA reported guidelines for the standards of bioanalytical method validation protocol. The StarDrop in silico software package (version 6.6), which involves the DEREK and WhichP450 in silico modules, was used to check the DVB structure for hazardous alerts and metabolic instability. The DVB and encorafenib (EFB), internal standard, and chromatographic peaks were successfully separated using a reversed phase column (an Eclipse Plus Agilent C8 column) and an isocratic mobile phase. The production of DVB parent ions was accomplished by utilizing the positive ionization mode of an ESI source. The identification and measurement of DVB daughter ions were conducted using the MRM mode. *Results*: The inter-day accuracy and precision exhibited a spectrum of values in the range of −0.56% to 9.33%, while the intra-day accuracy and precision showcased a range of scores between 0.28% and 7.28%. The DVB calibration curve showed a linear relationship that ranged from 1 to 3000 ng/mL. The usefulness of the currently validated UHPLC-MS/MS method was approved by the lower limit of quantification (LLOQ) of 1 ng/mL. The AGREE findings demonstrate that the UHPLC-MS/MS method had a noteworthy degree of ecological greenness. The in vitro half-life (t_1/2_) and intrinsic clearance (Cl_int_) of DVB were calculated to be 15.48 min and 52.39 mL/min/kg, respectively, which aligned with the findings from the WhichP450 software (version 6.6). *Conclusions*: Via the usage of in silico software, it has been observed that making small changes to the structure of the aryl piperazine ring and quinolinone moieties, or replacing these groups in the drug design process, shows potential for enhancing the metabolic safety and stability of newly developed derivatives compared to DVB.

## 1. Introduction

**Figure 1:**
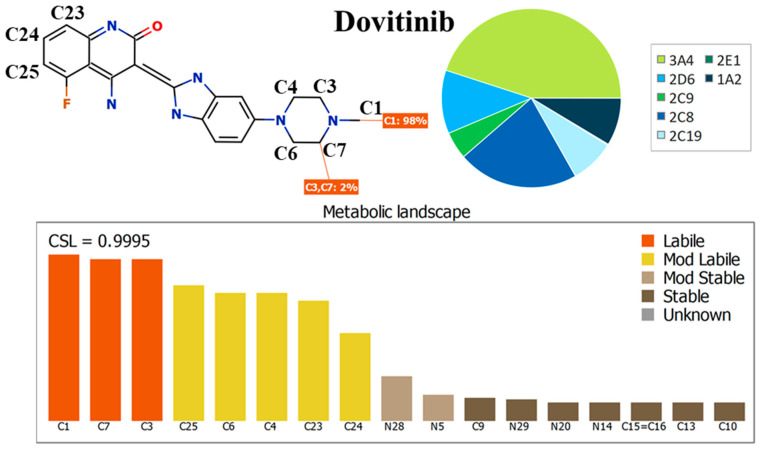
CSL (0.9995) proving the increase in the metabolic lability of DVB. The results were assessed using the WhichP450 module.

A cancerous tumor is defined by the unrestrained proliferation of malignant cells and the ability to extend to different regions inside the human body. Dovitinib (DVB), shown in Figure 1, is a benzimidazole-quinolinone molecule that inhibits several growth factor receptor kinases. It is also known as CHIR-258 or TKI258, and is accessible in an orally digestible lactate salt form [1,2,3]. The drug response predictor (DRP) biomarker algorithm was developed as a companion diagnostic to DVB (DRP-Dovitinib) [4]. Allarity uses its drug-specific DRP^®^ to choose patients who, by the genetic signature of their cancer, are found to have a high possibility of responding to the selected drug. By testing patients before treatment, the response rate can be considerably increased. DRP^®^ is based on messenger RNA from the patient’s biopsies. DVB has been granted FDA approval through a premarket approval application for a companion test (DRP-Dovitinib) in patients with renal cell carcinoma (RCC) [5,6].

DVB is a broad-spectrum tyrosine kinase inhibitor (TKIs) that targets various proteins that are commonly overexpressed in osteosarcomas and other bone sarcomas. These proteins include fibroblast growth factor receptor (FGFR), vascular endothelial growth factor receptor (VEGFR), and other receptor tyrosine kinases (RTKs). DVB is in clinical trials for several cancers, such as gastrointestinal stromal tumors [7], wild-type advanced urothelial carcinoma [8], and multiple myeloma [1]. In 2022, Oncoheroes obtained the exclusive license for dovitinib in the field of pediatrics from Allarity Therapeutics. In September 2023, the US FDA awarded the Orphan Drug Designation (ODD) to DVB for its application in the treatment of relapsed or advanced pediatric solid tumors, specifically osteosarcoma [9].

The main target of the current experiment is to estimate the metabolic stability of DVB in a laboratory context, utilizing a newly established UHPLC-MS/MS approach. Moreover, the gathering of additional data was made easier by using DEREK and WhichP450 software. The in silico StarDrop software, which combines the DEREK and WhichP450 modules, was used to estimate the metabolic stability and detect probable structural alarms in the DVB chemical structure. The determination of computational metabolic stability was performed using the StarDrop software (version 6.6), with specific emphasis on the WhichP450 program. The term “lability” in the framework of DVB metabolism refers to the assessment of the value of the production stage in the enzymatic cycle of the CYP3A4 enzyme. The estimation of the composite site lability (CSL) value can be used to determine the extent of metabolic lability. A reduction in CSL is linked to a higher possibility of improved stability. The CSL value was utilized as a first sign to provide the value of establishing in vitro operations, such as the creation of chromatographic methods and in vitro metabolic incubation studies, to optimize time control and resource distribution [10]. The Derek software (KB 2018 1.1) was utilized to monitor the incidence of hazardous alerts in the DVB chemical structure, so as to offer evidence for the hypothesized concept of in silico metabolic stability.

The creation of a rapid, green, and responsive analytical UHPLC-MS/MS technique for quantifying DVB in diverse substances holds great significance. The accurate assessment of a particular medication (DVB in the present study) is important for therapeutic drug monitoring (TDM) [11,12,13]. Moreover, it is essential to get an understanding about the correlation between the activity of DVB and its concentration level to guarantee the consistent and safe utilization of this drug by patients. An extensive analysis of the updated literature reveals a distinguished lack of reported research on the measurement of DVB in various matrices. Currently, there is an absence of published articles on the evaluation of the metabolic stability of DVB in HLMs utilizing an UHPLC-MS/MS methodology. The determination of the metabolic stability of DVB in HLMs is vital for understanding the kinetics of its metabolic reactions and elimination [14].

The stability of a pharmaceutical drug to metabolic enzymes refers to its susceptibility to undergo metabolic processes, and is estimated by its intrinsic clearance (Cl_int_) and in vitro half-life (t_1/2_) [15,16]. The phrase “half-life” denotes the time required for the 50% metabolism of the DVB. The intrinsic clearance (Cl_int_) generally refers to the hepatic capacity to metabolize the drug (DVB in the current investigation). It is a term used to designate enzyme activity, and does not depend on hepatic blood flow and protein binding [17]. The objective of this work was to develop a fast, selective, ecologically green, and sensitive UHPLC-MS/MS method for determining the metabolic stability of DVB in HLMs. The data’s validity was verified by doing in vitro testing using HLMs incubations and performing in silico analyses with the help of the StarDrop software package. Green analytical chemistry (GAC) has gained increasing significance in recent years. Its objective is to indicate the presence of dangerous chemicals, reduce waste production, and reduce energy consumption through diverse analytical approaches [18,19,20]. Different measurement methods have been used to determine the degree of ecological sustainability depending on different analytical discoveries, with the aim of accomplishing these objectives. The collection includes a series of methodologies known as the Analytical Green-ness Metric Approach (AGREE), Analytical Eco-Scale (AES), Green Analytical Procedures Index (GAPI), the National Environmental Methods Index (NEMI), and Red-Green-Blue (RGB) [18]. The GAPI, AES, RGB, and NEMI approaches show a reliance on specific GAC values, as explained. The study employed the “AGREE” approach to determine the level of ecological sustainability by considering 12 Greenness Assessment Criteria (GAC) and conveying values to each criterion [21].

The UHPLC-MS/MS method employed a constant mobile phase and had a running time of 1 min, representing an exceptionally rapid analytical technique. Introducing a flow rate of 0.5 mL/min and the use of low ACN% (45%) significantly enhanced the ecologically friendly features of the existing system. Furthermore, the methodology employed in the current research showed a linear relationship in the range from 1 to 3000 ng/mL. The verification of the importance of the necessity to develop an UHPLC-MS/MS approach for estimating DVB in HLMs matrix was achieved by undertaking software assessments of the metabolic stability of DVB, employing the WhichP450 module (StarDrop’s software) before beginning with the incubation of DVB with HLMs [10,22,23,24]. The current strategy was introduced with the aim of minimizing time and reducing costs. The current research used the UHPLC-MS/MS technology to estimate the Cl_int_ and in vitro t_1/2_ of DVB, as previously described in other investigations [25,26,27,28]. These methods can be utilized to compute the metabolic rate in living organisms using three different models: parallel tube, dispersion, and venous equilibrium. An in vitro technique was used to estimate the t_1/2_ and Cl_int_ of DVB, based on a well-stirred model [29,30]. The usage of this model is mostly noted in research on drug metabolism due to its simplicity and straightforwardness of execution.

## 2. Materials and Methods

### 2.1. Materials

The solvents utilized in the UHPLC-MS/MS method in the present research were of HPLC grade. The solid compounds, namely, DVB and encorafenib (EFB), were purchased in analytical research grade. The current investigation centers on the examination of two distinct analytes, specifically dovitinib (CHIR-258) and encorafenib (LGX818). The analytes were obtained from MedChemExpress, a well-regarded pharmaceutical company (Princeton, NJ, USA). The purity of DVB (Cat. No.: HY-50905) was found to be 99.94%, but EFB (Cat. No.: HY-15605) demonstrated a purity of 99.63%. Acetonitrile (ACN; Product No. 34851), ammonium formate (NH_4_COOH; Product No. 70221), HLMs, and formic acid (HCOOH; Product No. 5.33002) were derived from Sigma Aldrich Corporation, located in St. Louis, MO, USA. The HLMs (Product No. M0317) were transported using dry ice to guarantee the maintenance of their integrity. The HLMs (20 mg/mL) were stored in a refrigerated environment at −78 °C upon their arrival until they were considered appropriate for use.

### 2.2. Instruments

The water filtration method that was used to produce HPLC-grade water employed the Milli-Q apparatus developed by Millipore Corporation, located in Billerica, MA, USA. The investigation utilized the Acquity TQD MS (QBB1203) and the Acquity UPLC (H10UPH), both of which are analytical equipment produced by Waters Corporation. The instruments are categorized as an UPLC-MS/MS system. The instrument was used to conduct a comprehensive analysis and identify the chromatographic peaks in the substances (DVB and EFB) after they were extracted (via protein precipitation) from the HLMs incubation matrix. The UPLC-MS/MS apparatus was employed, utilizing MassLynx (Version 4.1, SCN 805) as the selected operating software. The vacuum in the TQD detection analyzer was initially created utilizing a vacuum pump manufactured by Sogevac Corporation (Murrysville, PA, USA). Nitrogen gas was utilized to improve the evaporation of mobile phase droplets in the ESI source. The gas was generated using a nitrogen generator obtained from Peak Scientific Company (Scotland, UK).

### 2.3. Assessment of DVB Metabolic Lability

Before running the incubation of DVB with active HLMs, the metabolic stability of DVB was assessed using WhichP450 in silico software that was developed by Optibrium Ltd. in Cambridge, Massachusetts, USA. The efficacy of performing in vitro incubations was validated using the outcomes acquired via the StarDrop software module. The results underwent further examination using a CSL approach, resulting in significant conclusions about the metabolic stability of DVB. The inclusion of the CSL feature was crucial in determining the DVB metabolic stability before initiating the in vitro HLMs incubation. The target of this step was to validate the importance of implementing the UHPLC-MS/MS approach for estimating the DVB metabolic stability. By incorporating the SMILES notation into the metabolic protocol, it became possible to evaluate the metabolic stability of DVB. Scientists collected data on the reactivity of individual atoms and consequently utilized this information to estimate the CSL value. Therefore, this specific value yielded valuable information about the metabolic instability of DVB. The CSL was computed using Equation (1).

(1) CSL=ktotal(ktotal+kw)

where k_w_ is the rate constant of water formation.

### 2.4. Screening of the DVB Toxicity Alerts Using DEREK In Silico Software

The evaluation of probable toxicity for DVB was done utilizing the DEREK module. In addition, the in silico software was used to detect structural alerts linked with DVB, with the target of proposing chemical structural changes that could alleviate the identified toxicity [31,32].

### 2.5. DVB In Silico ADME Profile

The SwissADME in silico software, established by the Swiss Institute of Bioinformatics that is located in Lausanne (Switzerland), was employed to forecast the ADME characteristics of DVB. The software can be used via the web platform at http://www.swissadme.ch/. The online software was accessed on 15 December 2023 [20,33,34].

### 2.6. UHPLC-MS/MS Analytical Features

The UHPLC-MS/MS features were adjusted to provide the highest sensitivity and resolution of the DVB and EFB analytical peaks, as listed in Table 1. The chromatographic qualities of the HPLC technology were enhanced through a comprehensive analysis of several factors, involving mobile phase, the pH level, and the stationary phase features. The objective of this optimization experiment was to enhance the resolution and sensitivity of the peaks related to the objectives DVB and EFB, as listed in Table 1. The isocratic mobile phase consisted of two components—line A, which consisted of an aqueous solution (0.1% HCOOH in H_2_O) at pH of 3.2, making up 55% of the mobile phase; and line B, which consisted of ACN, making up 45% of the mobile phase. The measured flow rate was found to be 0.5 mL/min. If the pH value reached around 3.2, the use of a 10 mM NH_4_COOH in H_2_O caused the appearance of a chromatographic peak tailing in the DVB analysis, as well as a longer running time. If the concentration of acetonitrile (ACN) surpasses 45%, the analytical chromatograms of DVB and EFB exhibit noticeable overlapping peaks. Conversely, a lower % of ACN generates a prolonged running time. The ESI was functioned in the positive ionization mode to aid ionization, as the DVB and EFB targets contained nitrogen atoms that could catch protons, generating positively charged ions.

The optimization of MS features for DVB (C_21_H_21_FN_6_O) and EFB (C_22_H_27_ClFN_7_O_4_S) was successfully performed using the IntelliStart^®^ software, a smart software component of the MassLynx 4.1 software package (version 4.1, SCN 805), by directly introducing DVB and EFB (10 µg/mL) inside the mobile phase stream. The mass-resolving power of MRM was used for the quantitative estimation of DVB and EFB. The implementation of this technique resulted in a higher degree of sensitivity and specificity for the UHPLC-MS/MS methodology that was devised. The ions of interest, specifically DVB and EFB, underwent fragmentation in the fragmentation quadrupole (collision cell), generating their corresponding product ions. The dissociation process was improved by employing argon gas (collision gas) with a high level of purity (99.999%). The measured dwell period for the conversion of mass from parent ions to daughter ions in DVB and EFB was found to be 0.025 s. The acquired outcomes were scrutinized and evaluated utilizing the QuanLynx program. Table 2 lists a comprehensive summary of the various parameters associated with MRM and mass transition in DVB and EFB.

### 2.7. DVB and EFB Working Dilutions

The maximum solubility of DVB and EFB was observed in DMSO at levels of 25 mg/mL (63.71 mM; attained by using both warming and ultrasonic treatment) and 50 mg/mL (92.59 mM; achieved solely through ultrasonic treatment), respectively. Therefore, the DVB and EFB stock solutions (1 mg/mL) were prepared in DMSO. The mobile phase was tuned to prepare working solutions of DVB at 100, 10, and 1 µg/mL, and EFB at a concentration of 220 µg/mL, using a gradual dilution method.

### 2.8. DVB Calibration Levels

Before initiating the validation phase for the UHPLC-MS/MS approach, the HLMs matrix was made inactive by adding DMSO (2%) and incubating it at 50 °C for 5 min. The incorporation of this precautionary estimate targeted alleviating the probable metabolic special effects of HLMs [35,36,37] on the analytes under investigation, specifically DVB and EFB. A validation matrix was specifically created for HLMs studies to evaluate the metabolic stability of DVB. The experimental steps included adding 30 µL of inactivated HLMs, which had a protein at 1 mg/mL, to 1 mL of metabolic matrix. The metabolic buffer solution was formulated by combining 0.1 M sodium phosphate at a pH of 7.4. In addition, the buffer solution contained 3.3 mM of MgCl_2_ and 1 mM of NADPH. The objective of utilizing this technology was to simulate the features of genuine in vitro incubation for practical experiments. The DVB (WK2 and WK3) underwent a series of dilutions using the inactive HLMs matrix in order to create DVB calibration standards, also known as CSs. Therefore, a grand total of eight CSs were generated at 1 to 3000 ng/mL. Additionally, four quality controls (QCs) were formulated, as follows: 2400 ng/mL (the high QC, HQC), 900 ng/mL (the medium QC, MQC), 3 ng/mL (the lower QC, LQC), and 1 ng/mL (LLOQ). During the multi-step dilution process, strict methods were followed to maintain the HLMs matrix at a level exceeding 90%. The target of this activity was to decrease the possible influences of matrix dilution, thereby reproducing the conditions of in vitro incubation. The QCs were utilized as unknowns, and their values were determined by applying the regression equation generated from the parallel injection of DVB CSs. An internal standard solution of EFB WK at 20,000 ng/mL was added into 100 µL to 1 mL of all DVB CSs and QCs.

### 2.9. The Extraction Recovery of DVB and EFB

The protein precipitation method was used for the extraction of the DVB and EFB from the HLMs matrix. The ACN organic solvent was used in this process to hinder and precipitate proteins included in the HLMs. Thus, ACN (2 mL) was used for each DVB CSs and QCs. Afterwards, the mixture was continuously agitated for 5 min to increase the extraction efficiency of the DVB and EFB from the deposited proteins. Then, the mixture was subjected to centrifugation at 4 °C and 14,000× rpm for 12 min. The centrifugation process was utilized to separate proteins and get a clear state in the supernatant. A filtration process was applied to all incubates to assess the appropriateness of the samples used in the LC-TQD equipment. The process employed the use of a 0.22 µm syringe filter. The filtered extracts were injected into a UHPLC-MS/MS system. To carry out the experiment, 2 control samples were established. The negative control sample was composed of HLMs. The positive control sample was composed of HLMs, which were enriched with EFB. The aforementioned approaches were reproduced to verify that the contents of HLMs did not have any influence on the separation process of DVB and EFB. The procedure for creating a calibration curve for a DVB included graphing the provided DVB scores on the x-axis, with the y-axis representing the peak area ratio of DVB to EFB. The range of the DVB CS’s linearity was evaluated by analyzing the validation parameters and the linear regression equation (y = ax + b; r^2^).

### 2.10. Validation Features of the Established UHPLC-MS/MS Approach

The validity of the UHPLC-MS/MS approach was assessed following the validation guidelines specified by the US FDA. The validation tactic included assessing many reported parameters, involving accuracy, precision, stability, linearity, extraction recovery, specificity, sensitivity, and matrix effect [38,39].

#### 2.10.1. Specificity

The specificity of the UHPLC-MS/MS approach was evaluated by injecting 6 groups of blank HLMs samples after precipitating the proteins, which was chosen as the preferred extraction method. The purified extracts were introduced into UHPLC-MS/MS equipment for analysis. The purpose was to identify any probable interference peaks that might arise from the HLMs matrix, occurring at the same elution time as the analytical peaks (DVB or the IS, EFB). Subsequently, a comparative study was conducted to assess the collected data with the spiked HLMs samples including DVB and EFB. The usage of the MRM analyzer detection method was employed to eliminate the residual effects of the DVB and EFB in the TQD detector. The validation was achieved via the study of the outcomes attained from the negative control samples of HLMs, which showed inadequacies in DVB and EFB.

#### 2.10.2. Sensitivity and Linearity

The evaluation of the sensitivity and linearity of the UHPLC-MS/MS system included the loading of 12 calibration curves. The creation of these curves entailed employing eight CSs for DVB, which were performed within the HLMs matrix, all accomplished in a single day. Subsequently, the regression equation of the constructed calibration curve that was created was utilized to determine the DVB in samples of unknown origin. The LOD and LOQ were determined following the ICH guidelines [40]. The study’s guidelines entail estimating the LOD and the LOQ by utilizing the slope and the SD of the intercept, as provided by Equations (2) and (3) [41].

(2) LOD=3.3∗SD of the interceptSlope

(3) LOQ=10∗SD of the interceptSlope

The determination of the linearity in the present UHPLC-MS/MS approach included the use of statistical measures, specifically the least squares methodology (y = ax + b) and the correlation coefficient (r^2^).

#### 2.10.3. Accuracy and Precision

The evaluation of accuracy and precision in the UHPLC-MS/MS approach required doing various experiments within one day for intra-day analysis and over the course of three following days for inter-day analysis. For the inter-day study, a total of six groups of DVB QCs were utilized. On the other hand, the intra-day investigation utilized twelve groups of DVB QCs. The determination of the UHPLC-MS/MS method’s precision and accuracy involved measuring the %RSD and %E, respectively. The calculation of these values was done using Equations (4) and (5), respectively.

(4) %Error=(Mean conc. − proposed conc.)proposed conc.∗100

(5) %RSD=SDMean

#### 2.10.4. Extraction Recovery and Matrix Effect

The determination of the HLMs’ influence on the generation of DVB or EFB ions was performed by forming two separate groups of samples. HLMs were used to test samples from group 1. The HLMs were enriched with the DVB at 3 ng/mL (LQC). In addition, an IS (EFB) was included into the solution at 2000 ng/mL. Instead, group 2 substituted the HLMs utilizing the mobile phase. The normalized matrix effect (ME) for the IS was estimated following Equation (6) [42], while the MEs for DVB and EFB were estimated utilizing Equation (7) [43,44].

(6) IS normalized ME=ME of DVBME of EFB(IS)

(7) ME of DVB or EFB=Average peak area ratioGroup 1Group 2×100

The assessment of the extraction recovery of DVB from the HLMs and the estimation of the influence of HLMs on the extent of DVB parent ionization were performed by the injection of 4 QCs. The efficacy of precipitating proteins as the optimized extraction methodology for DVB and EFB was assessed by administering six groups of four QCs samples in the HLMs matrix (B), and subsequently comparing them with four QCs produced in the chosen mobile phase (A). The assessment of recoveries for DVB and EFB including estimating the percentage by multiplying the result of dividing B by A by 100.

#### 2.10.5. Stability

This study sought to evaluate the stability of DVB in an HLMs matrix and stock solutions utilizing different laboratory circumstances, involving pre-analysis procedures such as long-term storage, short- term storage, three freeze–thaw cycles, and storage in an auto-sampler.

### 2.11. Evaluation of the System Greenness Employing AGREE Software

The determination of the overall ecological sustainability, or environmental integrity, of the supposed UHPLC-MS/MS technology was performed using the computational software (AGREE). The program has been designed to encompass all twelve metrics established by the GAC community [18]. The in silico software employs a weighting technique that assigns values between 0.0 and 1.0 to various components of the GAC approach.

### 2.12. In Vitro Assessment of the DVB Metabolic Stability

The evaluation of Cl_int_ and in vitro t_1/2_ of DVB included measuring the amount of DVB that remained after performing in vitro metabolic incubation. The incubation protocol was performed by applying the HLMs matrix, which was initiated with MgCl_2_ and NADPH as a coenzyme for the metabolic reaction. The in vitro incubation research was conducted using a four-step methodology. During the initial stage, a 1 µL sample of DVB was pre-incubated with HLMs. The above-mentioned methodology was performed with a water bath maintained at 37 °C through thermostatic management for 10 min. At the beginning of the experiment, each sample was given a solution containing 1 mM NADPH. Subsequently, all samples were kept in a shaking water bath at 37 °C. In the third step of the study, 100 µL of EFB (2000 ng/mL) was added prior to ACN addition, which acted as a quenching agent. The target of this step was to attain a uniform IS level and minimize any possible influence of metabolic enzymatic reactions on the IS concentration. During the fourth step, known as the cessation phase, a 2 mL sample of ACN was added at certain time points (0, 2.5, 7.5, 15, 20, 25, 30, 45, and 60 min) to quench the metabolic pathway and remove any excess proteins through precipitation. The initial step of the extraction methodology for DVB and EFB is denoted as the primary phase, as outlined in Section 2.9. An HLMS negative control was prepared to investigate the influence of omitting NADPH during the incubation of DVB with HLMs, following the previously reported approach. The target of the performed experiment was to estimate the possible effects of incubation sets and HLMs’ ME on the DVB concentration in realistic in vitro metabolic incubation experiments.

The residual DVB level was estimated by using the equation of the regression line generated from the calibration curve of DVB. The DVB metabolic stability curve was built by graphing the specified time intervals (x-axis) ranging from 0 to 60 min against the % DVB that remained compared to the DVB at time zero (100%) (y-axis). Therefore, the portion of the metabolic curve between 0 and 25 min was chosen to produce a logarithmic curve. The time span of 0–25 min, as previously mentioned, was used to plot the LN of DVB levels against the relevant time intervals. To get the rate constant for DVB metabolic stability, the slope of the previous graph was analyzed. Afterwards, the slope was used to estimate the in vitro t_1/2_ using the formula in vitro t_1/2_ = ln2/slope. The estimation of the Cl_int_ in mL/min/kg was performed by consulting prior research [45]. The computation in question entailed the usage of the matrix mass of HLMs (45 mg) per gram of liver tissue and the liver tissue mass (26 g) per kilogram of body weight, as given in Equation (8) [46].

(8) $${Cl}_{int,}=0.693\times \frac{1}{t_{½}(min.)}\times \frac{mL\;incubation}{mg\;protein}\times \frac{mg\;microsomal\;proteins}{g\;of\;liver\;weight}\times \frac{g\;liver}{Kg\;b.w.}$$

## 3. Results

### 3.1. In Silico Metabolic Lability Assessment of DVB

The CSL value of 0.9995 (Figure 1) provides an empirical indication for the substantial metabolic lability of DVB. Metabolic instability was revealed to be significant at C1 of the N-methyl group (98%), and at C3 and C7 of the piperazine ring (2%). A moderate level of instability was discovered at C4 and C6 of the piperazine ring, as well as at C23, C24, and C25 of the quinolinone moiety. CSL revealed the piperazine ring (Figure 1) as the main component that may significantly affect the metabolic instability of DVB. The CSL score of 0.9995 indicates a strong likelihood for metabolism. The above-mentioned findings align with the outcomes of further in vitro metabolic research, which will be clarified in the upcoming results. The metabolic stability of DVB was determined by applying the UHPLC-MS/MS system following a metabolic incubation experiment with HLMs.

### 3.2. In Silico Testing of DVB Toxicity Alerts Using DEREK Module

**Figure 2:**
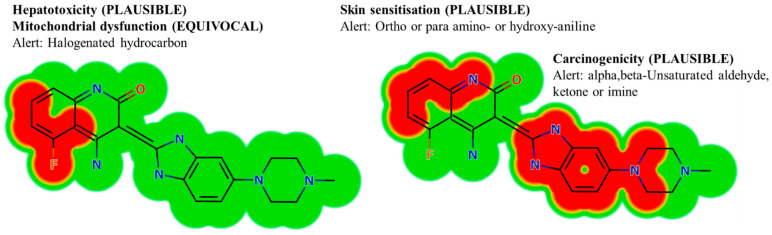
Structural alarms of DVB using DEREK in silico toxicity prediction tool of the StarDrop software (toxicity alerts marked in red color).

The occurrence of halogenated hydrocarbon is linked to the prediction of clear signs of liver toxicity (PLAUSIBLE) and the uncertain impairment of mitochondrial function (EQUIVOCAL). Alpha, beta-unsaturated ketone or imine is linked to structural signs of carcinogenicity (PLAUSIBLE). The presence of ortho-, para amino- or hydroxy-aniline may plausibly lead to skin sensitization. The previous studies have exhibited that metabolic instability and toxicity can be linked to the aryl piperazine ring and quinolinone moiety. This agrees with the results derived from computational evaluations of metabolic stability. Modifying the stated part or changing the group in the drug design context can improve the safety profile and metabolic stability of newly developed compounds in comparison to DVB (Figure 2).

### 3.3. In Silico ADME Parameters

**Figure 3:**
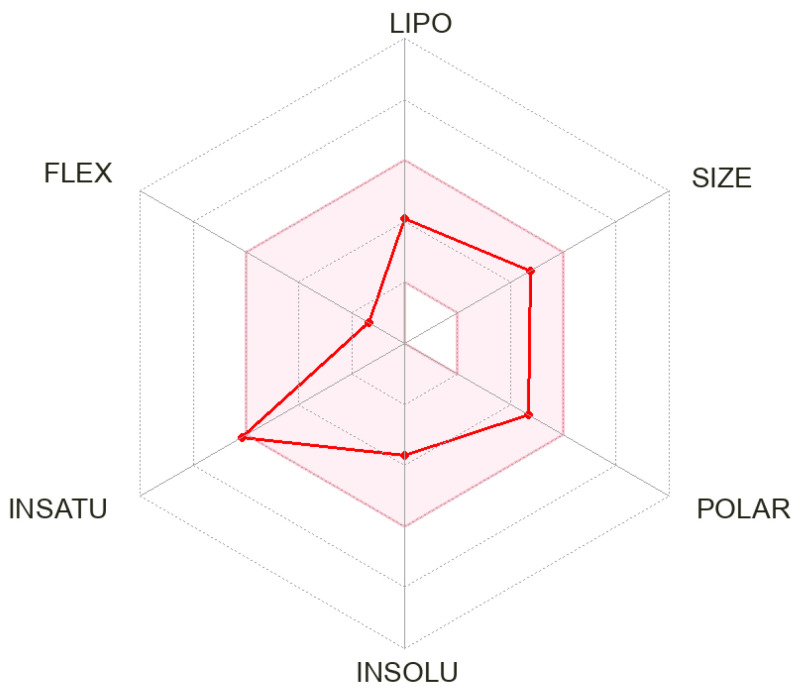
The ADME radar chart of DVB generated from the freely available SwissADME online software.

The SwissADME algorithm calculated that the water solubility of DVB had a desirable characteristic, as indicated by a log p value of −3.766. Furthermore, it is significant to mention that the expected pharmacokinetic features related to the absorption process in the gastrointestinal tract (GIT) show a significant rise, whereas there was no observed permeability through the BBB. The indicated bioavailability value is 0.55. DVB is expected to act as a suppressor for specific cytochrome P450 enzymes, namely, CYP2D6 and CYP1A2, in addition to P-glycoprotein, which acts as a substance that is transported. The proposition states that DVB does not demonstrate inhibitory effects on other cytochrome P450 enzymes, such as CYP3A4, CYP2C9, and CYP2C19. The Log Kp value, indicating skin permeability, is measured as −7.53 cm/s. Regarding druglikeness, it adheres to the Ghose, Lipinski, Egan, Muegge, and Veber criteria. Figure 3 exhibits the ADME radar chart for DVB, while Table 3 shows the equivalent information.

### 3.4. UHPLC-MS/MS Approach

Some stationary phases were evaluated, including HILIC columns. Neither the DVB nor the EFB were resolved or retained. Utilizing a C8 column has demonstrated advantageous outcomes as the reversed stationary phase. Conversely, when using the UHPLC-MS/MS approach to resolve DVB and EFB, the use of a C18 column causes the analytes to be retained in the chromatographic system. However, the observed targets, specifically DVB and EFB, with longer tails of the peak, exhibited inadequate distinctions of the main peak, and a prolonged elution period. The use of an Agilent C8 column (Eclipse plus), characterized by specific parameters such as a 50 mm length, a 3.5 μm particle size, and a 2.1 mm inner diameter, produced favorable results in terms of elution time and the shapes of analytical peaks. Table 4 shows a thorough compilation of several experiments performed to improve and determine the best characteristics for extracting, separating, and evaluating DVB and EFB peaks. The main goal of these experiments was to achieve a desirable feature, such as an acceptable peak shape and a reduced running duration.

**Figure 4:**
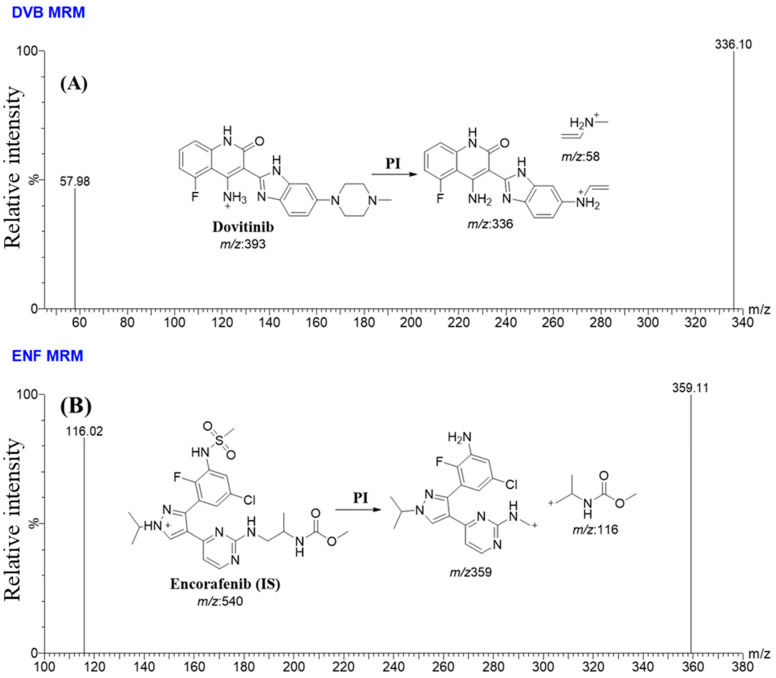
MRM (two mass transitions) mass spectrum of DVB [M + H]^+^ (**A**) and Encorafenib [M + H]^+^ (**B**) represented as two mass transitions. The proposed dissociation behaviors are elucidated.

So as to enhance the specificity and sensitivity features of the UHPLC-MS/MS instrument, the MRM analyzer mode was put in place to accurately detect and measure the levels of DVB and EFB. The target of this research was to investigate any possible interference produced by the HLMs matrix ingredients (Figure 4).

**Figure 5:**
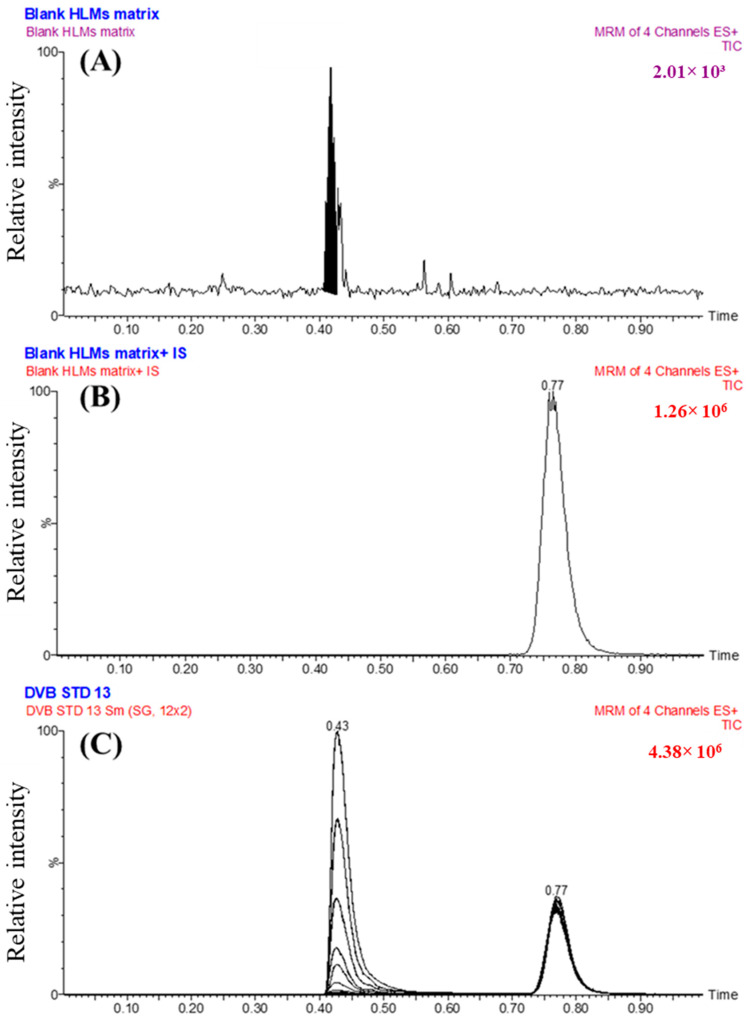
Negative control HLMs showed no interfering chromatographic peaks at the retention times of DVB and ENF (**A**). The total ion chromatogram (TIC) and MRM chromatogram of HLMs (negative control) with ENF at 2000 ng/mL (**B**). The overlaid MRM mass chromatograms of the 8 DVB CSs (**C**). The chromatograms reveal the peaks conforming to DVB (at 0.43 min) and IS at 2 µg/mL and at an elution time of 0.77 min.

The MRM chromatograms of the negative control (Figure 5A) and positive control (Figure 5B) for DVB in the HLMs did not show any carry-over impact. Figure 5C shows the mass chromatograms obtained from the MRM analysis of DVB CSs and EFB. The range of concentrations covered is from 1 to 3000 ng/mL for DVB CSs and 2000 ng/mL for EFB.

### 3.5. Validation of the LC-MS/MS Approach

#### 3.5.1. Specificity

The efficacy of the UHPLC-MS/MS method was proven by its effective separation of analytical peaks associated with DVB and EFB, as displayed in Figure 5. Furthermore, the investigation exhibited that the analytical peaks of DVB and EFB were not significantly affected by the components of the HLMs matrix. No visible carry-over effect resulting from the preceding sample analysis performed using DVB was noticed in the MRM chromatograms of the negative and positive controls in the MS analyzer (MRM) mode analysis.

#### 3.5.2. Linearity and Sensitivity

**Figure 6:**
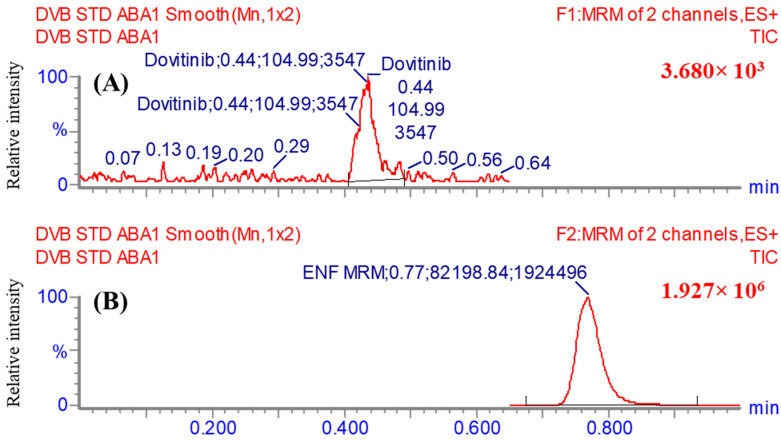
DVB LOQ peak at 1 ng/mL (**A**). Additionally, the EFB (IS) peak at 2000 ng/mL (**B**).

Statistical analysis was utilized to prove the linear behavior of the current UHPLC-MS/MS system, covering the linearity range 1–3000 ng/mL. An extremely high degree of correlation was seen between the variables, with an r^2^ value of 0.9997 and a regression equation of y = 1.035x + 0.8911. Because of the wide CSs range (1 to 3000 ng/mL), a weighting factor of (1/x) was included in the building of the DVB linear calibration curve [47]. Table 5 reveals that the RSD of the six repetitions, involving CSs, was below 7.28%. Figure 6 illustrates the confirmed LOQ at 1.0 ng/mL.

#### 3.5.3. Accuracy and Precision Validation Parameters

The acquired data of accuracy and precision were judged to show the intended acceptable values, as reported in the validation guidelines established by the FDA [48]. The inter-day accuracy and precision exhibited a spectrum of scores from −0.56% to 9.33%, while the intra-day accuracy and precision showcased scores in the range of 0.28% to 7.28% (Table 6).

#### 3.5.4. The HLMs Matrix Does Not Show Any Observed Effect on the Extraction Recovery of DVB in the Proposed UHPLC-MS/MS System

The outcomes of the investigation show a significant level of recovery for DVB extraction (102.62 ± 3.73% with an RSD of less than 3.63%) and EFB (101.61 ± 3.23% with an RSD lower than 3.18%). The HLMs matrix, consisting of DVB and EFB, had an ME of 102.39 ± 4.64% for DVB and 99.74 ± 3.85% for EFB, respectively. The normalized ME of the IS was computed to be 1.03, which is within the acceptable limit stated by the FDA’s regulatory criteria. The study’s results suggest that there is no statistically significant correlation among the HLMs matrix and the level of parent ionization for both EFB and DVB.

#### 3.5.5. Stability of DVB in the HLMs (The Metabolic Incubation Matrix) and DMSO

The stability assessment of DVB in HLMs and DMSO matrices reveals that the optimal stability was achieved by storing the DVB in DMSO at −80 °C for 28 days. The relative standard deviation (RSD%) for all samples of DVB consistently stayed below 4.13% across all storage conditions, as shown in Table 7. There was no considerable decrease in the DVB level observed after subjecting it to different stability parameters. The results reported in this study provide supporting evidence for the high level of consistency demonstrated by the DVB.

### 3.6. Evaluation of the UHPLC-MS/MS System Greenness Utilizing the AGREE Software

**Figure 7:**
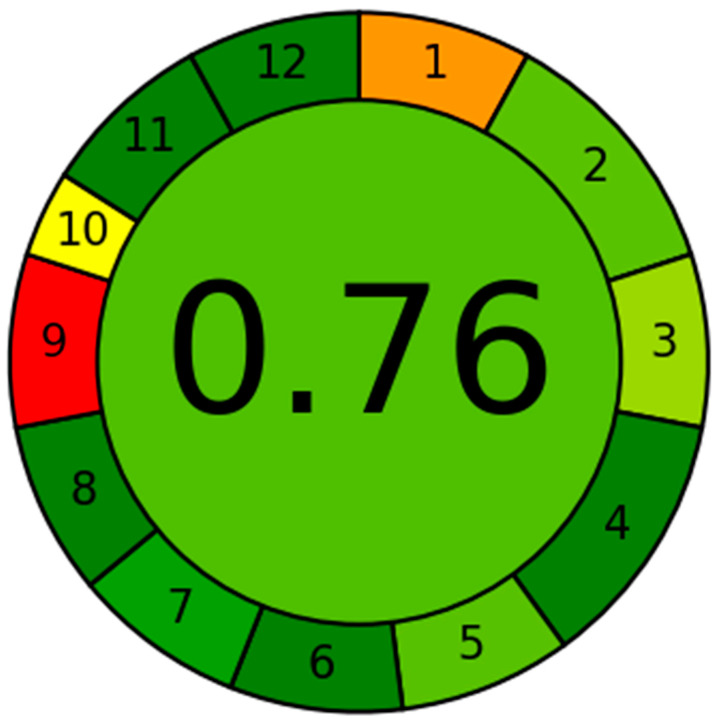
The AGREE in silico software was utilized to assess the eco-friendly profile of the current UHPLC-MS/MS technique displayed in the shape of a circular diagram of 12 distinct traits.

The GAC approach produces analytical scales that are highly effective in evaluating the sustainability degree. The data are displayed as a circular pattern that involves a diverse variety of colors, spanning from dark green to red, indicating twelve distinct traits. Figure 7 shows the extent to which the UHPLC-MS/MS methodology demonstrates ecological sustainability. The measured values for each of the 12 qualities were then gathered and are shown in Table 8. The current methodology was assessed based on multiple criteria, yielding a score of 0.77. The score quantifies the level of ecological responsibility obtained by implementing the UHPLC-MS/MS system. A value near 1.0 reveals a better degree of sustainability in the analytical methodology. The current UHPLC-MS/MS system, a newly developed method, has a noteworthy degree of environmental accountability, as revealed by an eco-scale value (0.77) ranging from 0.75 to 1.00.

### 3.7. In Vitro Metabolic Incubations of DVB with HLMs

**Figure 8:**
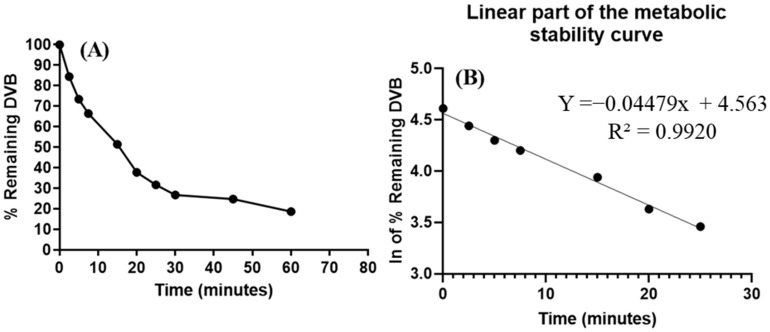
(**A**) DVB metabolic stability curve; (**B**) the natural logarithm (ln) curve (linear segment) exhibiting the linear regression equation.

The negative control showed no noteworthy change in DVB level. With the aim of determining the metabolic stability of DVB, an experimental level of 1 µM/mL of DVB was used in the in vitro studies utilizing active HLMs matrix. The choice to utilize this particular level was made to keep the level under the Michaelis–Menten constant. This establishes a clear association among the metabolic rate of DVB and the incubation time of in vitro HLMs (Figure 8). HLMs at 1 mg protein/mL were utilized to mitigate non-specific protein binding. The research findings reveal that the rate of metabolism (slope) of the DVB was calculated to be 0.0383, as evidenced by the equation y = −0.04479x + 4.563, and displayed a high r^2^ of 0.992 (Table 9). The in vitro t_1/2_ was 15.48 min. The clearance of DVB was determined to be 52.39 mL/min/kg.

## 4. Discussions

The current UHPLC-MS/MS approach involves separating the required analytes, DVB and EFB, using a mobile phase solution with a constant composition. The resolution operation was performed at 0.5 mL/min for one minute. The calibration curve for the DVB, as estimated utilizing the disclosed approach, showed a linear correlation across the whole range of 1 to 3000 ng/mL.

The usage of EFB as an IS in the quantification of DVB was implemented following the UHPLC-MS/MS methodology, which depends on three crucial criteria. The protein precipitation extraction technique is proven to be effective in extracting both DVB and EFB targets, yielding 101.28 ± 2.84% with an RSD below 2.80% for DVB, and 101.61 ± 3.23% with an RSD below 3.18% for EFB. Furthermore, the peaks of DVB (0.43 min) and EFB (0.77 min) were attained in a one-minute timeframe. The aforementioned outcome provides proof of the success of the UHPLC-MS/MS method as a proficient and efficient analytical instrument. The used method not only efficiently decreases the overall period of the procedure, but also optimizes the utilization of ACN, hence lining up with the principles of sustainable chemistry. Also, it is essential to recognize that the participants in a particular medical setting did not take both substances (DVB and EFB) at the same time. Hence, the UHPLC-MS/MS approach can be applied for pharmacokinetic and TDM experiments on DVB.

The in silico metabolic lability (0.9995) aligned with the in vitro metabolic stability investigation of DVB with HLMs. The metabolic stability of DVB was determined by applying the UHPLC-MS/MS system following a metabolic incubation experiment with HLMs. The in vitro t_1/2_ was 15.48 min. The clearance of DVB was determined to be 52.39 mL/min/kg. Following McNaney et al. [45], DVB is classified as a drug with high clearance in the grading system. This means that it can be given devoid of any doubts about the accumulation of dosages in the human body. Utilizing in silico software, such as simulation and Cloe PK software, can provide vital data about the DVB in vivo pharmacokinetics, thereby uncovering important physiological variables [49].

**Figure 9:**
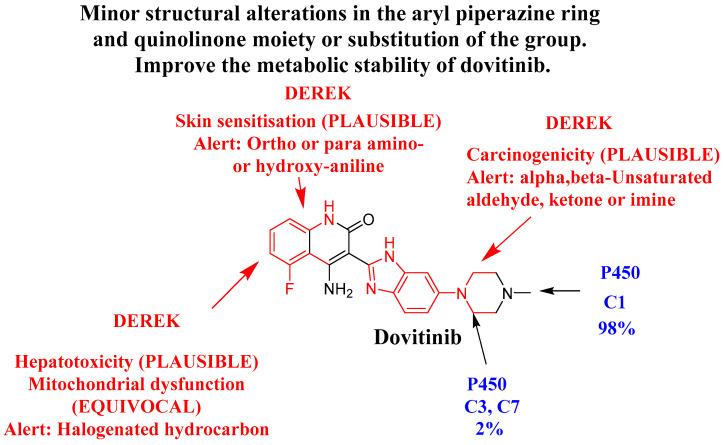
Dovitinib’s metabolic lability (blue color) using WhichP450 software. Dovitinib DEREK toxicity assessments (red color) revealing that the aryl piperazine ring and quinolinone moiety are accountable for the proposed toxicity and metabolic instability of DVB.

Following the data derived from the in silico DEREK and WhichP450 softwares, it is proposed that creating small changes to the structure of the aryl piperazine ring and quinolinone moieties, or replacing theses structural alerts in the drug design process, might possibly improve the stability properties and metabolic safety of new derivatives, in comparison to DVB (Figure 9). Modifying the stated part or changing the group in the drug design context can improve the safety profile and metabolic stability of newly developed compounds in comparison to DVB [50,51].

## 5. Limitation of the Current Study

The developed UHPLC-MS/MS approach was validated only in the HLMs matrix for metabolic stability estimations. For the widespread use of this analytical method, it should be revalidated in different biological fluids. The results derived using the in silico software are not conclusive. They need to be confirmed by synthetizing the proposed chemical structures through changing the proposed moieties and testing their metabolic stability and pharmacological action.

## 6. Conclusions

This study involved developing and evaluating a UHPLC-MS/MS method to measure DVB levels in HLMs. Subsequently, the previously mentioned method was utilized to quantify the in vitro metabolic stability of DVB. The UHPLC-MS/MS system showed a few notable features, including the ecological compatibility, increased sensitivity and selectivity, and good recovery of DVB and EFB from the HLMs matrix. The outcomes were produced by using ACN to precipitate proteins as the chosen extraction approach. The UHPLC-MS/MS approach was designed with a specific interest in incorporating environmentally friendly practices using the targeted processes. The methods involved utilizing a low flow rate of 0.5 mL/minute, reducing the amount of ACN to 45%, and shortening the analysis period to 1 min. After performing an assessment of the ecological sustainability utilizing the AGREE software, it may be stated that the UHPLC-MS/MS methodology has ecologically friendly features and is potentially better suited to the routine estimation of DVB in other biological fluids after revalidation for the new matrix, without any detrimental influence on the environment. The data attained from the computational investigation of the WhichP450 metabolism using StarDrop’s in silico software were confirmed utilizing in vitro incubation studies with HLMs. The acquired data on metabolic stability, including a t_1/2_ of 15.48 min and a high Cl_int_ of 52.39 mL/min/kg, suggest that the DVB exhibits features that are in line with those of a drug with a high extraction ratio. Possible avenues for future work could be revealed by using the current approach, which includes the usage of in silico software tools and laboratory-based metabolic incubations. The utilization of these methodologies is important for the development of novel pharmaceutical development, most notably in enhancing metabolic stability. The benefits of using a variety of computer-based software methods to preserve resources and decrease effort are shown in the data attained from the screening software and laboratory studies using HLMs of DVB.

## Figures and Tables

**Table 1:** UPLC-MS/MS tuned parameters.

UPLC			TQD MS		
Eclipse plus-C8 reversed column	Particle size	3.5 μm	ESI	Positive ESI source	
	Internal diameter	2.1 mm		The extractor voltage	3.0 (V)
	Length	50.0 mm		Cone gas rate	100 L/hr
	Column T	22.0 ± 2.0 °C		The RF lens voltage	0.1 (V)
Isocratic mobile phase system	Aqueous part	0.1% Formic acid in H_2_O		Nitrogen gas	Drying gas
		55%			100 L/hr
		pH: 3.2			350 °C
	Organic part	45% ACN		Capillary voltage: 4 KV	
	Injection volume:	5.0 μL	Argon gas	0.14 mL/min	
	Flow rate	0.5 mL/min.	Mode	MRM	

**Table 2:** MRM features for the quantitative estimation of DVB and EFB.

	Time Segment	Rt	Mass Transitions (*m*/*z*)
MRM detection segments	0.0 to 0.65 min	DVB (0.44 min)	393→336 (CE ^a^:32 and CV ^b^: 28)
			393→58 (CE:36 and CV: 46)
	0.65 to 1.0 min	EFB (IS; 0.77 min)	540→359 (CE: 56 and CV: 36)
			540→116 (CE: 56 and CV: 32)

^a^ Dissociation energy, ^b^ the voltage of the cone.

**Table 3:** ADME characteristics of DVB derived by the freely available online SwissADME software.

Physicochemical Properties		Water Solubility	
Formula	C_21_H_21_FN_6_O	Solubility	8.60 × 10^−2^ mg/mL; 2.19 × 10^−4^ mol/L
Molecular weight	392.43 g/mol	Log S (ESOL)	−3.66
Heavy atoms num.	29	Class	Soluble
Arom. heavy atoms num.	19	Solubility	2.32 × 10^−1^ mg/mL; 5.92 × 10^−4^ mol/L
Rotatable bonds num.	2	Log S (Ali)	−3.64
Fraction Csp3	0.24	Class	Soluble
		Solubility	8.66 × 10^−5^ mg/mL; 2.21 × 10^−7^ mol/L
Num. H-bond donors	3	Log S (SILICOS-IT)	−6.66
TPSA	94.04 Å^2^	Class	Poorly soluble
Num. H-bond acceptors	4	**Medicinal Chemistry**	
Molar refractivity	120.28	Brenk	0 alert
**Lipophilicity features**		PAINS	0 alert
Log Po/w (XLOGP3)	1.64	Leadlikeness	No; 1 violations: MW > 350
Log Po/w (iLOGP)	2.26	Synthetic accessibility	3.20
Log Po/w (MLOGP)	2.31	**Pharmacokinetics**	
Log Po/w (SILICOS-IT)	3.27	GI absorption	High
Log Po/w (WLOGP)	2.21	P-gp substrate	Yes
Consensus Log Po/w	2.34	Permeant to BBB	No
**Druglikeness features**		Inhibiton of CYP1A2	Yes
Ghose	Yes	Inhibiton of CYP2D6	Yes
Lipinski	Yes; 0 violation	Inhibiton of CYP3A4	No
Egan	Yes	Inhibiton of CYP2C9	No
Veber	Yes	Inhibiton of CYP2C19	No
Muegge	Yes	Skin permeation (Log Kp)	−7.53 cm/s
The score of bioavailability	0.55		

**Table 4:** Tuned UHPLC-MS/MS parameters.

Analytes	Recovery		Stationary System		Stationary System	
	Solid Phase Extraction	Protein Precipitation Using ACN	Methanol	ACN	C18 Column	C8 Column
DVB	Low (88.27%)	High (102.62 ± 3.73%)	0.52 min	0.43 min	0.74 min	0.43 min
	Not precise	Precise (RSD < 3.63%)	Tailed	Good peak	Tailed peaks	Perfect shape
EFB	Good (89.89%)	High (101.61 ± 3.23%	0.87 min	0.77 min	1.25 min	0.77 min
	Not precise	Precise (RSD < 3.18%)	Overlapped	Optimum peak shape	Perfect shape	Optimum shape

**Table 5:** Back-calculation data of six replicates (CSs) of DVB.

DVB (ng/mL)	Mean	SD	Accuracy (%)	RSD (%)	Recovery
1.00	1.07	0.08	7.18	7.28	107.18
15.00	14.73	0.09	−1.83	0.64	98.17
40.00	40.93	0.28	2.32	0.69	102.32
100.00	100.96	1.57	0.96	1.56	100.96
250.00	254.41	1.90	1.77	0.75	101.77
500.00	508.02	5.97	1.60	1.18	101.60
2000.00	1966.90	21.08	−1.65	1.07	98.35
3000.00	2997.38	13.60	−0.09	0.45	99.91
% Recovery					101.28 ± 2.84

**Table 6:** Intra-day and inter-day validation features (accuracy and precision values) of the established UHPLC-MS/MS system.

DVB (ng/mL)	Intra-Day (12 Sets in 1 Day)				Inter-Day (6 Sets in 3 Days)			
QCs	1.00	3.00	900.00	2400.00	1.00	3.00	900.00	2400.00
Mean	1.07	3.10	902.61	2406.71	1.09	3.05	894.99	2389.57
SD	0.08	0.03	7.54	10.10	0.03	0.06	5.18	5.74
Precision (%RSD)	7.28	0.81	0.83	0.42	2.30	2.00	0.58	0.24
% Accuracy	7.18	3.19	0.29	0.28	9.33	1.68	−0.56	−0.43
Recovery (%)	107.18	103.19	100.29	100.28	109.33	101.68	99.44	99.57

**Table 7:** DVB stability features.

Stability as Validation Features	Mean		SD		Precision (%RSD)		Accuracy (%E)	
	3.0	2400.0	3.0	2400.0	3.0	2400.0	3.0	2400.0
Long-Term (−80 °C for 28 d)	2.91	2388.44	0.05	5.52	1.82	0.23	−3.00	−0.48
Auto-Sampler (15 °C for 24 h)	3.01	2406.68	0.12	7.77	4.13	0.32	0.22	0.28
Freeze–Thaw (3 cycles at −80 °C)	3.06	2414.45	0.08	14.51	2.72	0.60	2.11	0.60
Short-Term (4 h at room T)	2.95	2390.13	0.11	6.38	3.59	0.27	−1.56	−0.41

**Table 8:** The UHPLC-MS/MS approach report sheet following the GAC guidelines.

Principles	Score	Weight
1. To avoid the necessity for sample treatment, it is recommended to use direct analytical methods.	0.3	2
2. The targets of this research are to achieve a minimal amount and a small sample size of specimens.	0.75	3
3. Ideally, it is advisable to perform assessments on-site whenever it is feasible.	0.66	2
4. Studies have shown that the integration of analytical steps and activities leads to positive results concerning energy preservation and reducing the use of reagents.	1.0	3
5. It is advisable to choose automated and streamlined operations.	0.75	2
6. Avoiding the implementation of derivatization procedures is prudent.	1.0	2
7. To reduce the causing of a noteworthy amount of analytical surplus and implement effective disposal methods is paramount.	0.88	2
8. Within the field of analytical chemistry, there is a predilection for employing multi-analyte or multi-parameter methodologies as opposed to those that just concentrate on a single analyte.	1.0	2
9. Attempts should be considered to reduce energy use.	0.0	2
10. It is sensible to give importance to the usage of reagents produced from renewable sources.	0.5	1
11. The necessity of eliminating or replacing detrimental substances is of paramount significance.	1.0	2
12. There is a necessity to improve the safety regulations for workers.	1.0	2

**Table 9:** The parameters of dovitinib (DVB) metabolic stability.

Time (min.)	Mean ^a^ (ng/mL)	X ^b^	LN X	The Linear Segment Features
0.00	380.45	100	4.61	Regression line equation: y = −0.04479x + 4.563
2.50	321.44	84.49	4.44	
5.00	279.33	73.42	4.30	R^2^ = 0.9920
7.50	252.96	66.49	4.20	
15.00	195.86	51.48	3.94	Slope: −0.04479
20.00	143.62	37.75	3.63	
25.00	120.75	31.74	3.46	t_1/2_: 15.48 min and
30.00	101.88	26.78	3.29	Cl_int_: 52.39 mL/min/kg
45.00	94.28	24.78	3.21	
60.00	71.30	18.74	2.93	

^a^ Average of 3 repeats. ^b^ X: Average of the % residual of DVB in 3 repeats.

## Data Availability

The original contributions presented in the study are included in the article; further inquiries can be directed to the corresponding author.
